# In Vitro Probiotic Modulation of the Intestinal Microbiota and 2′Fucosyllactose Consumption in Fecal Cultures from Infants at Two Months of Age

**DOI:** 10.3390/microorganisms10020318

**Published:** 2022-01-29

**Authors:** Alicja M. Nogacka, Silvia Arboleya, Naghmeh Nikpoor, Jeremie Auger, Nuria Salazar, Isabel Cuesta, Jorge R. Alvarez-Buylla, Laura Mantecón, Gonzalo Solís, Miguel Gueimonde, Thomas A. Tompkins, Clara G. de los Reyes-Gavilán

**Affiliations:** 1 Department of Microbiology and Biochemistry of Dairy Products, Instituto de Productos Lácteos de Asturias (IPLA-CSIC), 33300 Villaviciosa, Asturias, Spain; silvia.arboleya@ipla.csic.es (S.A.); nuriasg@ipla.csic.es (N.S.); icuesta@ipla.csic.es (I.C.); jorge.rodriguez@ipla.csic.es (J.R.A.-B.); mgueimonde@ipla.csic.es (M.G.); 2 Institute of Health Research of the Principality of Asturias (ISPA), 33011 Oviedo, Asturias, Spain; laura_mantecon@hotmail.com (L.M.); GSOLIS@telefonica.net (G.S.); 3 Rosell^®^ Institute for Microbiome and Probiotics, Montreal, QC H4P 2R2, Canada; naghme.nikpoor@gmail.com (N.N.); jeremieaauger@gmail.com (J.A.); ttompkins@lallemand.com (T.A.T.); 4 Pediatrics Service, Central University Hospital of Asturias (HUCA-SESPA), 33011 Oviedo, Asturias, Spain

**Keywords:** *Bifidobacterium bifidum*, *Bifidobacterium longum* subsp. *infantis*, *Lactobacillus helveticus*, probiotic formulation, 2′-fucosyllactose, microbiota, in vitro model, infants

## Abstract

2′-fucosyllactose (2′FL) is one of the most abundant oligosaccharides in human milk, with benefits on neonatal health. Previous results point to the inability of the fecal microbiota from some infants to ferment 2′FL. We evaluated a probiotic formulation, including the strains *Lactobacillus helveticus* Rosell^®^-52 (R0052), *Bifidobacterium longum* subsp. *infantis* Rosell^®^-33 (R0033), and *Bifidobacterium bifidum* Rosell^®^-71 (R0071), individually or in an 80:10:10 combination on the microbiota and 2′FL degradation. Independent batch fermentations were performed with feces from six full-term infant donors of two months of age (three breastfed and three formula-fed) with added probiotic formulation or the constituent strains in the presence of 2′FL. Microbiota composition was analyzed by 16S rRNA gene sequencing. Gas accumulation, pH decrease and 2′FL consumption, and levels of different metabolites were determined by chromatography. *B. bifidum* R0071 was the sole microorganism promoting a partial increase of 2′FL degradation during fermentation in fecal cultures of 2′FL slow-degrading donors. However, major changes in microbiota composition and metabolic activity occurred with *L. helveticus* R0052 or the probiotic formulation in cultures of slow degraders. Further studies are needed to decipher the role of the host intestinal microbiota in the efficacy of these strains.

## 1. Introduction

Microbial colonization of the infant gut is a key process that starts in utero during gestation, increases considerably after birth, and influences the maturation of the immune system, the development of the neonatal intestine and general physiology, and later the health of infants [1]. Human milk oligosaccharides (HMOs) are a diverse group of oligosaccharides that are abundant in human milk. 2′-fucosyllactose (2′FL) is one of the major HMOs present in human milk, and the ability of women to produce 2′FL varies during lactation and is controlled by genetic factors [2]. HMOs are able to promote the selective growth of some specific members of the intestinal microbiota, such as *Bifidobacterium* and *Bacteroides* species [3,4]. HMO consumption and the ability of the infant’s microbiota to metabolize these oligosaccharides have been strongly correlated with fecal microbiota composition in early life [5,6]. In this way, we have recently shown that the feeding mode (breast vs. formula) and the intrinsic ability of the microbiota of two-month-old infants to degrade 2′FL are determinant factors influencing the evolution of microbiota composition and metabolic activity in fecal cultures [7].

Although breastfeeding is considered the gold standard of nutrition for infants, some circumstances make it difficult, and the infants may require other forms of lactation such as the use of donated human milk from other mothers (whenever possible) and/or feeding with commercial milk formulations [8,9]. Introducing probiotics in commercial milk preparations may contribute to the correct establishment of the intestinal microbiota, and a wide variety of commercial preparations include probiotics in their formulation [9,10]. Prebiotics, such as fructooligosaccharides (FOSs) and galactooligosaccharides and, less frequently, HMOs, are also often added to commercial formula milk in order to improve the beneficial action of probiotics [10,11]. However, little is known about the effect of the combined use of probiotics and HMOs on the microbiota of newborns.

Strains from the *Bifidobacterium* genus and *Lactobacillus* group are the most widely used probiotics. Some of their species are Generally Recognized As Safe (GRAS status) by the Food and Drug Administration (FDA) in the USA or have been granted the Qualified Presumption of Safety (QPS status) by the European Food Safety Authority (EFSA). Lactobacilli, although representing a small proportion of the human gut microbiota, have been considered relevant members associated with health [12]. Bifidobacteria are among the first colonizers of the infant gut and one of the predominant members of its microbiota until weaning and transition to solid foods [13]. The most prevalent *Bifidobacterium* species in the infant gut are *Bifidobacterium breve*, *Bifidobacterium bifidum*, and *Bifidobacterium longum* [14]. Strains of *B. bifidum*, *B. longum* subsp *infantis*, and *B. breve* seem to be especially well adapted to metabolize HMOs [1,15,16].

Recently, the FDA concluded that the addition to milk-based infant formulas of the probiotic strains *Lactobacillus helveticus* Rosell^®^-52 (R0052), *B. longum* subsp. *infantis* Rosell^®^-33 (R0033), and *B. bifidum* Rosell^®^-71 (R0071), individually or in an 80:10:10 blend, at levels up to 3 × 10^9^ CFU/serving, is safe, granting to these microorganisms or their combination the GRAS status (GRAS notice 758, 2018). The efficacy in clinical studies with pediatric populations of this probiotic formulation with added FOS, commercialized in several countries under the names Biostime^®^ and Probiokid^®^, has been recently reviewed [17]. Moreover, the *L. helveticus* R0052 strain has proven to have beneficial effects at the level of the intestinal mucosa by hindering the access of pathogens to epithelial cells [18,19], whereas the probiotic formulation reinforces the modulating effect of this strain on the immune response to pathogens [20,21]. This, together with the convenience of producing industrially an economically affordable product with good microbiological viability, provides the rationale for the composition of this commercial probiotic multi-strain formulation. However, there is currently a gap of knowledge regarding the interaction of this probiotic mixture with the intestinal microbiota of newborns and the role of 2′FL on this interaction.

In a previous work, we studied the influence of 2′FL on fecal microbiota composition and metabolic activity by using in vitro fecal cultures of full-term vaginally delivered infants of two months of age fed exclusively breastmilk or formula [7]. The aim of the present study is to go a step forward by assessing the use of 2′FL together with the multi-strain probiotic formulation indicated above or their constituent strains as a synbiotic combination on the fecal microbiota of the same donors that participated in the previous study. The age of two months has been chosen as representative of the evolving microbiota of newborns—when a stable microbial pattern has not yet been established, and the high variability existing between neonates is still present.

## 2. Materials and Methods

### 2.1. 2′-Fucosyllactose Commercial Preparations and Probiotic Strains

Three commercial preparations of 2′FL were used and prepared as indicated elsewhere [7]. One of the preparations was from DuPont Nutrition and Health (2′FL-A), with a 93% purity in 2′FL; Aequival^®^ was from Friesland Campina Ingredients (Paramus, NJ, USA; 2′FL-B) and displayed 87.1% purity, whereas the third preparation was from Jennewein Biotechnologie GmbH (Rheinbreitbach, Rheinland-Pfalz, Germany; 2′FL-C) and had 87.8% purity in 2′FL.

Three strains (*L. helveticus* Rosell^®^-52: strain R0052; *B. bifidum* Rosell^®^-71: strain R0071; and *B. longum* subsp. *infantis* Rosell^®^-33: strain R0033) and a mixture of them in proportions 80:10:10, respectively, were used in the present work. Individual strains were re-activated from frozen stocks by two passages in MRS (De Man, Rogosa and Sharpe) medium (Biokar Diagnostics, Beauvois, France) supplemented with 0.25% (*w*/*v*) L-cysteine (MRSc; Sigma Chemical Co., St. Louis, MO, USA) and incubated for 24 h at 37 °C in an anaerobic chamber MG500 (Don Whitley Scientific, West Yorkshire, UK). The microbial suspensions for fecal cultures were obtained by inoculating fresh culture medium with the corresponding strain and incubated for about 5 h until an optical density (OD) of 1.3–1.7 for *L. helveticus*, 0.3 for *B. bifidum*, and 0.5 for *B. longum* subsp. *infantis* (exponential phase of growth) was obtained. Cultures were then centrifuged, washed twice with PBS (VWR Chemicals, Solon, OH, USA), and suspensions in PBS were adjusted to a final concentration of 2.4 × 10^9^ CFU/mL for *L. helveticus* and 3 × 10^8^ CFU/mL for *B. bifidum* and *B. longum* subsp. *infantis*.

### 2.2. Fecal Sample Collection and Batch Culture Fermentations

Fecal samples were obtained from six vaginally delivered full-term healthy children at two months of age. Three newborns were exclusively fed with breastmilk (BF) of their mothers and the other three received formula milk (FF) exclusively. Fecal samples from two of the BF infants were 2′FL fast degraders in fecal cultures and the other was a slow degrader, whereas, conversely, two fecal samples from FF donors were 2′FL slow degraders and one was a fast degrader [7]. Infants were recruited at the Neonatology Unit of Pediatric Service at the Central University Hospital of Asturias (HUCA, Northern Spain). All infants were discharged from the Hospital at 2–3 days of life. The study was approved by the Regional Ethical Committee of Asturias Public Health Service (n° 236/19), and informed written consent was obtained from each infant’s parents. Samples were collected at home over one or two weeks from nappies immediately after defecation using a sterile spatula and were frozen in sterile tubes at −20 °C until their transportation to the laboratory within the sample collection week. In an anaerobic chamber, a 1/5 (*w*/*v*) dilution of a minimum of 20 g of thawed fecal samples was carried out in pre-reduced PBS solution with 25% (*v*/*v*) glycerol, vortexed for 10 min, and stored in 20–30 mL aliquots at −80 °C until use.

Independent pH-uncontrolled fecal batch fermentations from the different infant donors were performed in the BMIF basal medium with added 5% (*v*/*v*) reconstituted formula milk (Novalac Initiation Formula 0–6 months, Paris, France) following manufacturer’s instructions, as described previously [7,22]. Culture conditions included 10 different combinations: each of the strains *L. helveticus* R0052, *B. longum* subsp. *infantis* R0033, *B. bifidum* R0071, and the multi-strain mixture of the three strains, alone or with each of the three 2′FL commercial preparations added, plus a negative control with no probiotic, alone or with 2′FL (Table S1). Among these combinations, those corresponding exclusively to cultures with 2′FL have been published elsewhere [7] and are used in the present work for comparison with the cultures supplemented with probiotics. Briefly, the pre-reduced medium was inoculated with the corresponding fecal homogenates (1%, *v*/*v*), distributed into bottles of the ANKOM RF system (Ankom Technology, Macedon, NY, USA), and stabilized in anaerobiosis [23]. Then, 2′FL preparations were added to the corresponding bottles at a final concentration of 0.2% (*w*/*v*), and bacterial suspensions, prepared in the day of the assay, were added at the final levels of 2.4 × 10^7^ cfu/mL for *L. helveticus* and 3 × 10^6^ for *B. longum* subsp. *infantis* and *B. bifidum* strains. Bottles with the nine different conditions and the negative control were incubated under anaerobiosis at 37 °C for 24 h. This incubation time was considered appropriate to reveal differences in the microbiota profile caused by growth in the presence of probiotics and 2′FL, without introducing biases due to a nutrient depletion caused by longer times of incubation. Samples (1 mL) were taken in duplicate at time 0 before incubation (basal conditions) and, after 24 h of incubation, were centrifuged at full speed for 15 min; pellets and supernatants were stored separately at −20 °C until analyses.

### 2.3. Microbiota Composition Analysis

The bacterial taxonomic composition of pellets from fecal batch cultures at 24 h of incubation was determined by 16S rRNA gene sequencing of the V3–V4 region, as described by Nogacka et al. [7]. In short, the QIAamp Fast DNA Stool Mini Kit (Qiagen; Düsseldorf, Germany) was used for genomic DNA extraction as per the manufacturer’s protocol, with some modifications. Extracted genomic DNA samples were amplified using 1X KAPA HiFi HotStart ReadyMix (Roche, cat # KK2802) with universal 16S primers (forward 5′-CCTACGGGNGGCWGCAG-3′ and reverse 5′-GACTACHVGGGTATCTAATCC-3′) [24]. Cycling conditions for the PCR amplicon were as follows: initial denaturation at 95 °C for 3 min followed by 25 cycles of 95 °C for 30 s, primer annealing at 55 °C for 30 s and primer extension at 72 °C for 30 s before a 5 min final extension at 72 °C. Commercially available 20 strains mock communities (ATCC, cat # MSA-1002-even mix and MSA-1003-staggered mix) were used as sequencing controls and water as no-template amplification control. PCR products were visualized on a 2% agarose precast E-Gel stained with SYBR Safe dye (Invitrogen cat # G72080). Amplicons were purified with Agencourt AMPure beads (Beckman Coulter, cat # A63881) following the Illumina 16S Metagenomic sequencing library’s preparation protocol. A second round of amplification using 5 µL of the purified amplicon PCR reaction as template was performed in 25 µL reactions containing 2.5 µL of each of the Nextera XT V2 primers sets A, B, and C (Illumina, cat # FC-131-2001, FC-131-2002, and FC-131-2003) and 1X KAPA HiFi ReadyMix. The same cycling conditions as the Amplicon PCR were used, except that only 8 cycles were necessary to attach the different index combinations used as tags to identify each sample during multiplexing. PCR reactions were again purified with AMPure beads before individual fluorescent quantification by Quant-iT PicoGreen dsDNA assay (Life Technologies, cat # P7589) with a Varioskan LUX microplate reader (ThermoScientific, cat # VL0000D0) with 485 nm excitation and 520 nm emission wavelengths. Volumes corresponding to 200 ng of each purified Index PCR reaction were pooled using an EpMotion 5075 liquid handling robot (Eppendorf), and this pool was quantified with a QuBit Broad Range assay (ThermoScientific, cat # Q32853; Waltam, MA, USA) following the manufacturer’s instructions. Finally, the diluted pool (amplicon size 599 bps) and PhiX mixture were loaded in a MiSeq Reagent 600 cycles v3 Kit cartridge (Illumina, cat # MS-102-3003) and processed for 2 × 300 cycles paired-end sequencing on a MiSeq instrument.

### 2.4. Gas and pH Monitorization

The pH of fecal cultures (time 0 and after 24 h of incubation) and the cumulative gas produced during the different fermentation conditions using the ANKOM RF system were determined as described previously [7,23].

### 2.5. Analysis of 2′Fucosyllactose, Lactose, Monosaccharides, and Organic Acids by HPLC

The consumption of 2′FL in fermentation bottles with this HMO added, variations in the levels of lactose and the monosaccharides glucose, galactose, and fucose, as well as organic acids formed during incubation (lactic, pyruvic, succinic, and formic) were analyzed by HPLC in all culture bottles, as indicated by Nogacka et al. [7]. Cell-free supernatants from cultures were filtered (0.45 µm), injected using an Alliance 2695 module injector, and separated by ion-exclusion chromatography through a column ICSep ICE-ION (Teknokroma Analitica, Barcelona, Spain). A PDA 966 photodiode array detector was used for the determination and quantification of organic acids, a 2414 differential refractometer detector for determination and quantification of carbohydrates (2′FL, lactose, glucose, fucose, and galactose), and Empower software (Walters, Milford, MA, USA) for identification and quantification of peak areas. Chromatographic and analysis conditions were those described elsewhere [25]. Results were expressed in mg/100 mL. Variations in the levels of the analyzed metabolites at 24 h of incubation were calculated for each fermentation batch, when appropriate, with respect to the basal conditions (time 0, ∆). The consumption of 2′FL at 24 h of incubation was calculated with respect to the concentration of 2′FL in basal conditions (time 0), considering this as 100%.

### 2.6. Analysis of Short-Chain Fatty Acids by Gas Chromatography

The analysis of short-chain fatty acids (SCFAs) was performed by gas chromatography (GC) in the fecal culture supernatants to quantify acetic, propionic, butyric acid (major SCFAs), as well as isobutyric and isovaleric acids, as described in Nogacka et al. [7]. Briefly, 250 µL of culture supernatants collected at time 0 and 24 h of incubation were mixed with 0.3 mL methanol, 0.05 mL of the internal standard solution (2-ethylbutyric acid 1.05 mg/mL), and 0.05 mL of 20% (*v*/*v*) formic acid. The mixture was centrifuged, and the supernatant was collected for SCFA quantification in a system composed of a 6890N GC injection module (Agilent Technologies Inc., Palo Alto, CA, USA) connected to a flame injection detector (FID) (Agilent). Samples were analyzed in triplicate, and results were expressed in µg/mL. Increments (∆) in the levels of these compounds at 24 h of incubation with respect to the basal conditions (time 0) were calculated for each fermentation batch with the different combinations of probiotics and 2′FL.

### 2.7. Bioinformatic Data Processing and Statistical Analyses of the Microbial Community

The fastq files of the 16S sequencing were imported into QIIME2 (Quantitative Insight Into Microbial Ecology–2) as artefacts and were analyzed as described previously [7]. In short, the quality filter software of QIIME2 was used to process the demultiplexed amplicons. The imported data set was inspected, the reads were trimmed at 240 base pairs, and the quality-filter q-score using the default parameters was employed for quality control. The reads were then clustered into amplicon sequence variants (ASVs) with the denoising algorithm Deblur of the QIIME2 suite [26]. The feature classifier was used to attribute the ASVs to the closest known taxa using QIIME2’s sk-learn classification module [27]. The taxonomy file (linking ASV sequences to known taxonomic groups) was trained on a 99% clustered GreenGenes database. The taxonomic profiles at the family level for each probiotic treatment with or without the addition of 2′FL were presented as stacked bar-plots for each of the four groups previously defined on the basis of the basal microbiota of donors to degrade 2′FL after 24 h (combinations of BF/FF and Fast/Slow 2′FL degraders) [7].

### 2.8. Statistical Analyses of Microbiota Composition and Microbial Metabolites

All experimental data are reported as mean ± standard deviation. Statistical analysis of results for the metabolic activity of fecal cultures was performed using the software SPSS v.26 (SPSS Inc., Chicago, IL, USA). Data from 2′FL consumption, pH evolution, gas production, and variations in the levels of lactose, monosaccharides, organic acids, and SCFA were compared in fecal cultures with the different probiotic treatments at the end of fermentation (24 h). The Kruskal-Wallis test was used to compare the effect caused on the microbiota composition and microbial metabolites by the addition of different probiotic strains and the probiotic formulation in combination with 2′FL and the experimental condition where only 2′FL was added (control). Data were analyzed in the four fecal culture groups previously defined on the basis of the ability of the basal microbiota to degrade 2′FL and the feeding type of fecal donors: BF-fast degraders (breastfed, 2′FL fast degraders, *n* = 2), BF-slow degrader (breastfed, 2′FL slow degrader, *n* = 1), FF-fast degrader (formula-fed, 2′FL fast degrader, *n* = 1), FF-slow degraders (FF, 2′FL slow degrader, *n* = 2) [7]. As the three 2′FL commercial preparations used in fecal cultures displayed similar fermentability, cultures with the different 2′FL formulations were considered triplicates for a given experimental condition with the same fecal inocula [7].

### 2.9. Nucleotide Sequence Accession Numbers

The raw sequences reported in this article were deposited in the NCBI Sequence Read Archive (SRA) under the accession number PRJNA731876.

## 3. Results

### 3.1. 2′-FL Degradation Profile in Fecal Cultures with Probiotics Added

**Figure 1:**
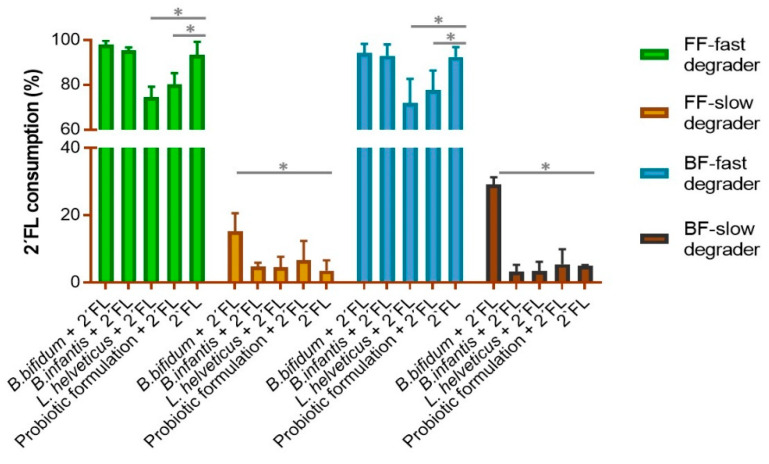
2′FL consumption (% of decrease in the levels of 2′FL from values at baseline, considered as 100%) by fecal cultures with added probiotics plus 2′FL with respect to fecal cultures only, with 2′FL added after 24 h of incubation for each of the four fecal culture groups previously defined for the same fecal inocula according to the feeding type of donors and intrinsic capacity of the basal microbiota to degrade 2′FL: FF-fast degrader, FF-slow degraders, BF-fast degraders, BF-slow degrader. Significant differences between 2′FL cultures and those with each added probiotic and the probiotic formulation plus 2′FL are indicated by asterisks over bars. * *p*-value < 0.05.

We assessed to which extent the addition of probiotics could modify the degradation profile of 2′FL in fecal cultures, as related to the intrinsic capacity of their microbiotas to degrade this HMO and the feeding type of donors [7]. In fecal cultures of fast degraders, regardless of the mode of feeding, the addition of bifidobacteria strains did not modify the degradation pattern of 2′FL (Figure 1). However, the presence of either the multi-strain probiotic mix formulation or the *L. helveticus* strain individually caused a significant reduction in the degradation of 2′FL, this effect being more pronounced in cultures with *L. helveticus* R0052 than with the probiotic formulation added. In contrast, in fecal cultures of slow degrader donors, the strain *B. bifidum* R0071 promoted an increase in the degradation of 2′FL, although the consumption rate of this HMO remained lower than in fecal cultures of fast degraders (Figure 1).

### 3.2. Effect of Probiotics and Their Combination with 2′FL on the Microbial Composition

**Figure 2:**
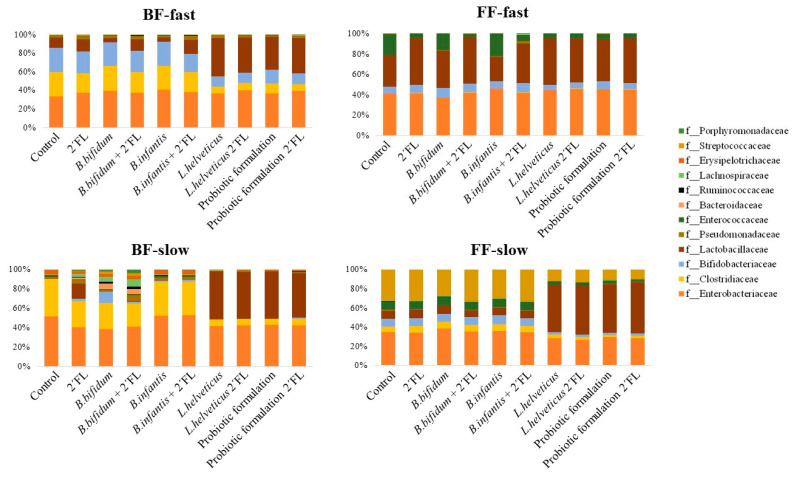
Relative abundance of bacteria at the family level at 24 h of incubation in fecal cultures with the different probiotics and the probiotic formulation added with and without 2′FL for each fecal culture classification group, ranked according to the feeding type of donors and the intrinsic capacity of the basal microbiota to degrade 2′FL [7].

Some variations in the microbiota composition profiles were found by 16S rRNA gene sequencing after the incubation of fecal cultures with the different probiotics added, in the presence or absence of 2′FL (Figure 2). Overall, at the family taxonomic level, changes seem to be more pronounced in cultures of slow degraders than in those of fast degraders regardless of the feeding type of donors. Moreover, *L. helveticus* R0052 added to fecal cultures at levels one log unit higher than bifidobacteria strains or the multi-strain probiotic formulation, alone or in synbiotic combination with 2′FL, promoted the increase of the relative abundance of the Lactobacillaceae family in most fecal cultures, a decrease of Clostridiaceae in cultures of BF donors (both fast and slow degraders) and of FF-slow degrader donors, and the decrease of the Streptococcaceae, Enterococcaceae and Enterobacteriaceae families in cultures of FF-slow degraders. Of note was the increase of the families Bacteroidaceae and Ruminococcaceae, together with the decrease of Enterobacteriaceae in fecal cultures from the BF-slow degrader donor, with added *B. bifidum* R0071, with and without 2′FL, and the increase of Bifidobacteriaceae with the same probiotic, which was much more pronounced in the absence of 2′FL. Apart from this, other discrete changes promoted by probiotic bifidobacteria strains in the absence of 2′FL, as compared to control cultures, include the increase of the relative abundance of Bifidobacteriaceae and the decrease of Lactobacillaceae by the addition of *B. bifidum* R0071 or *B. longum* subsp. *infantis* R0033 to fecal cultures of BF-fast degraders and the increase of Lactobacillaceae and decrease of Enterococcaceae in cultures of the FF-fast degrader baby with added *B. bifidum* R0071 and the opposite effect in the same fecal cultures by the addition of the *B. longum* subsp. *infantis* strain. Nevertheless, the lack of enough experimental replicates of fecal cultures performed in the absence of 2′FL precludes us from obtaining sound conclusions through the statistical treatment of data.

We then compared differences at the family level at 24 h of incubation between fecal cultures added with probiotics in combination with 2′FL with respect to cultures of fast and slow degraders only added with 2′FL. No statistically significant differences were found for the major fecal microbial groups analyzed (Bifidobacteriaceae, Lactobacillaceae, and Streptococcaceae) in cultures added with *B. bifidum* or *B. longum* subsp. *infantis* strains as compared to fecal cultures of the same infant donors only with 2′FL added (Table 1), with the only exception of a striking but non-statistical significant decrease of the Lactobacillaceae family in cultures from BF-slow degraders. In contrast, microbiota changes were more apparent with *L. helveticus* R0052 or the multi-strain probiotic formulation.

These probiotics promoted the increase of the relative abundance of the Lactobacillaceae family in all fecal cultures (statistical significance for FF-slow and BF-fast degraders) except those of FF-fast degraders, where this taxon reached similar relative abundances as in cultures without probiotics. Hence, for cultures of slow degraders, in those from FF babies, lactobacilli reached similar levels at 24 h as in cultures of fast degraders of the same mode of feeding (FF) only with 2′FL added, whereas in cultures of BF infants, this taxon clearly surpassed levels found in cultures of BF-fast degraders without probiotics. On the other hand, the relative abundance of the Streptococcaceae family in cultures of FF slow degraders resulted significantly reduced in the presence of the *L. helveticus* strain or the probiotic combination with respect to cultures with added bifidobacteria or without probiotics. Nevertheless, the addition of *L. helveticus* R0052 or the probiotic formulation caused a general decrease of the relative abundance of the Bifidobacteriaceae family that reached statistical significance in cultures of BF-fast and the slow degrading donors. Finally, some minor but differentially significant shifts were observed in the relative abundance of Lachnospiraceae, Ruminococcaceae, and Erysipelotrichaceae families by the addition of *Bifidobacterium* and *L. helveticus* strains or their combination, as depending on the mode of feeding of baby donors and the capacity of the microbiotas to degrade 2′FL degradation.

All these data evidence a modulation of the microbiota by probiotics, which was specific for each probiotic and depended on the intrinsic characteristics of the basal microbiota composition and its capacity for degrading 2′FL.

### 3.3. Effect of the Probiotic Treatment on the Microbial Metabolic Activity of Fecal Cultures with 2′FL Added

Gas production, pH, SCFA (acetic, propionic, butyric, isobutyric, and isovaleric acids), organic acids (lactic acid, pyruvic acid, succinic acid, formic acid), and ethanol are indicators of the microbial metabolic activity in fecal cultures. Concentrations of fucose in the culture medium after incubation provide information about the degradation activity towards 2′FL, whereas levels of lactose, galactose, and glucose provide information either about the microbial activity on 2′FL or on the lactose present in the culture medium directly [7]. SCFA, organic acids, and ethanol are mainly formed through microbial carbohydrate degradation, some of these compounds being important for human health [28].

Fecal cultures with the strain *L. helveticus* R0052 or the probiotic formulation added experienced a more pronounced decrease of pH than cultures only with added 2′FL (Table S2). In the case of cultures with bifidobacteria, the addition of *B. longum* subsp *infantis* R0033, but not of the *B. bifidum* strain, to cultures from the BF slow-degrading donor promoted a significantly more pronounced decrease of pH than the sole addition of 2′FL. Moreover, *L. helveticus* or the probiotic formulation tended to decrease gas production in all fecal culture groups, although the values obtained did not reach statistical significance (Table S2).

The strain *B. bifidum* R0071 promoted the accumulation of fucose in cultures of slow degraders, regardless of the mode of feeding of fecal donors, which, in the specific case of the BF cultures, also coincided with a slight accumulation of lactose (Figure S1). In cultures supplemented with *B. longum* subsp. *infantis* R0033, no remarkable variations were obtained for the sugars analyzed with respect to cultures only with added 2′FL, with the unique exception of a slightly more pronounced consumption of galactose in cultures from the BF slow-degrading donor (Figure S1). Nevertheless, the addition of *L. helveticus* R0052 or the probiotic combination promoted changes in the sugars’ profile, which were generally characterized by a higher consumption of galactose in all fecal culture groups and a significantly higher consumption of lactose in cultures of slow-degrading donors, regardless of the mode of feeding, as compared to cultures only with 2′FL (Figure S1).

**Figure 3:**
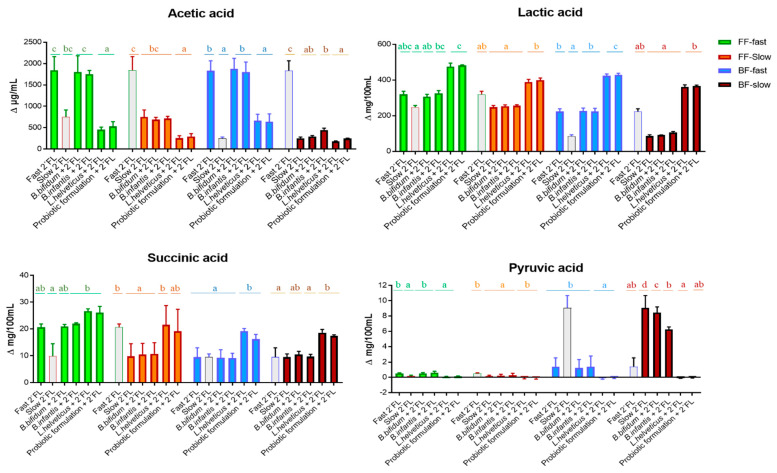
Variations after 24 h of incubation with respect to baseline (time 0) in the levels of microbial metabolites displaying any significant difference (acetic, lactic succinic, and pyruvic acid) between fecal cultures with 2′FL and added probiotics and the corresponding cultures only with 2′FL. Different letters on the horizontal lines above the bars indicate significant differences (*p* < 0.05) among the different conditions, analyzed separately in cultures of FF and BF infant fast and slow degraders. Bars shaded in grey correspond to cultures with different 2′FL velocities of degradation but the same mode of feeding as the group being analyzed and were considered for statistical comparisons with the group next to which they are placed in each case in the figure. Values for fast- and slow-degrading cultures with added 2′FL without probiotics have been published previously [7] and are used here for statistical comparisons.

Acetic and lactic acids were the major microbial metabolites formed in fecal cultures during incubation. *Bifidobacterium* strains did not promote significant variations in the production of these compounds. However, a lower increase of acetic acid levels and higher accumulation of lactic acid occurred generally due to the addition of *L. heveticus* R0052 or the probiotic formulation with respect to the corresponding cultures only with 2′FL added (Figure 3); notably, the accumulation of lactic acid in cultures with added *L. helveticus* or the probiotic combination exceeded the levels reached by fecal cultures of fast degraders only with 2′FL. Among the other microbial metabolites, no remarkable changes were found by the addition of the *Bifidobacterium* strains to fecal cultures, as compared to cultures only with 2′FL added (Figure 3; Table S2), with the exception of a significantly higher accumulation of pyruvic acid in cultures of the BF-slow degrader with respect to fast degraders; that, however, was attenuated to some extent in the presence of the probiotic *Bifidobacterium* strains. In contrast, important changes occurred in cultures with added *L. helveticus* R0052 and the probiotic formulation with respect to fecal cultures of the same donors only with 2′FL (Figure 3; Table S2). A significantly lower accumulation of pyruvic acid was found in all cases, which was concomitant with an increase of succinic acid and ethanol that reached statistical significance for succinic acid in cultures of BF babies and only with *L. helveticus* in cultures of FF-slow degraders. Remarkably, in all culture groups with the *L. helveticus* strain or the probiotic combination added, except in those from FF fast-degraders, the accumulation of succinic acid clearly surpassed the levels reached when only 2′FL was added (Figure 3).

Our data point to differential microbiota metabolic profiles after incubation in the presence of probiotics and 2′FL that were dependent on the specific probiotic and on the intrinsic capacity of the microbiota of fecal donors to degrade 2′FL.

## 4. Discussion

A probiotic formulation combining the strains *L. helveticus* R0052, *B. longum* subsp. *infantis* R0033, and *B. bifidum* R0071 with FOS has been commercialized in several countries for 20 years, and some clinical studies have reported positive health effects in pediatric populations [17]. However, little is known about the effect of these probiotics on the infant’s microbiota. In order to cover this gap of knowledge, preclinical studies with in vitro and in vivo models are affordable procedures before approaching more complicated and expensive human intervention studies. To this regard, no in vitro studies have been conducted until present about the effect of this formulation on the intestinal microbiota of infants. Therefore, the present study was performed to assess the effect of the multi-strain probiotic formulation just mentioned and each of their constituent microorganisms on the microbiota composition and metabolic activity in fecal cultures from full-term vaginally delivered babies at two months of age in the presence of one of the most abundant HMOs in human milk, the compound 2′FL. The ANKOM RF system was used to carry out in vitro fecal batch fermentations, as described recently [7]. The same fecal inocula, ranked previously in four groups according to the mode of feeding of infant donors (BF or FF) and the intrinsic capacity of their microbiotas to degrade 2′FL in fecal cultures (fast degraders and slow degraders), were considered in the present study and the results were analyzed according to this classification [7].

The addition of the strain *B. bifidum* R0071 to fecal cultures of slow-degrading donors, but not of any of the other strains or its combination, promoted the increase of 2′FL degradation during fermentation, reaching higher rates of consumption in cultures inoculated with feces from the BF baby than with those from FF donors. This evidenced the capacity of *B. bifidum* R0071 to promote the degradation of 2′FL, either by a direct action of this microorganism on 2′FL or through interactions with other microorganisms present in the infant microbiota. In this regard, several authors have reported the ability of *Bifidobacterium* strains from the species *B. bifidum* and *B. longum* to ferment 2′FL through cellular enzymatic mechanisms that are different between these two species [4,29,30]. FF and BF donors display different fecal microbiota compositions [7], and, unlike in other studies, bifidobacteria are not the most abundant bacteria in these babies [1,5,6]; differences in the basal microbiota composition could have accounted for differences in 2′FL degradation rates between cultures from both groups of feeding. Relating to this, Borewicz et al. [5] reported a strong correlation of HMO consumption with the fecal microbiota composition of BF infants at one month of age. In our work, the fact that cultures from slow-degrading donors with added *B. bifidum* R0071 reached lower degradation rates of 2′FL than fecal cultures of fast degraders suggests that in fast degraders, some other microorganisms of the microbiota may also be contributing to the efficient degradation of 2′FL together with the probiotic added. In this regard, some trophic interactions have been described between *Bifidobacterium* strains and other bifidobacteria or members of other intestinal microbial taxons for the efficient degradation of 2′FL [31,32]. Recently, Centanni et al. [33] demonstrated in co-cultures the syntrophic cooperation of two strains of *B. bifidum* (a 2′FL degrader not using fucose) and *B. breve* (a 2′FL non-degrader consuming fucose) for the joint use of 2′FL and its constituent sugars, resulting in modifications of the metabolism of both strains and of the growth pattern of *B. breve*.

We have recently reported a different evolution of the microbiota of infants in fecal cultures based on its intrinsic ability of the microbiotas to degrade 2′FL and on the feeding type of fecal donors [7]. In the current work, we analyzed the specific changes promoted by incubating these same fecal inocula in the presence of probiotics together with 2′FL and compared them to cultures of fast and slow degraders of the same mode of feeding with only 2′FL added. At 24 h of incubation, the addition of *B. bifidum* R0071 and *B. longum* subsp. *infantis* R0033 had not caused major specific changes in the abundance of the dominant microbial groups other than those obtained by incubation with 2′FL only. In spite of this, we found slight changes in the relative abundance of Lachnospiraceae and Ruminococcaceae families. This suggests that the addition of bifidobacteria strains could have modified the microbial dynamics of members of the fecal microbiota to a limited extent during fermentation. Moreover, the higher 2′FL degradation occurring in cultures with *B. bifidum* R0071 added promoted an increase of fucose accumulation in cultures of FF- and BF-slow degraders and of lactose in cultures of BF-slow degraders, not accompanied by remarkable variations in microbial metabolites produced as regards to cultures added only with 2′FL. This, together with the fact that the consumption of 2′FL did not reach the levels obtained in cultures of fast-degrading donors, supports the hypothesis that other microorganisms that are able to interact with *B. bifidum* R0071 for the efficient degradation of 2′FL are probably lacking in the microbiota of slow degrader donors. Supplementing infants displaying a 2′FL slow-degrading profile with probiotics that are able to efficiently degrade this and other HMOs in early life could positively contribute to the correct establishment of the intestinal microbiota in these infants; however, it is important to discard any potential harmful effect derived from the use alone of a particular HMO instead in concert with other HMOs [1].

Shifts in composition and metabolic activity of the microbiota due to the addition of the strain *L. helveticus* R0052 were much more pronounced than those promoted by the addition of *Bifidobacterium* strains, and this will be discussed next. The most noticeable changes in composition affected the microbial groups that we previously reported as becoming dominant during the incubation of fecal cultures in the presence of 2′FL [7]. Thus, a differentially marked increase of lactobacilli occurred at the expense of the decrease of the relative abundance of the Bifidobacteriaceae family in all fecal cultures except in those of FF-fast degraders where lactobacilli have been previously shown to become the dominant taxon after incubation in the presence of 2′FL [7]. Streptococcaceae, the taxon more abruptly increasing during incubation with 2′FL in FF-slow degraders, showed considerably decreased abundance after incubation when fecal cultures were also performed with added *L. helveticus* R0052. Overall, our results seem to indicate that *L. helveticus* R0052 promoted in cultures of BF-fast degraders a shift in the relative abundances of bifidobacteria and lactobacilli that move away from the optimal microbial profile of cultures from BF-fast degraders [7]. However, the same strain favored in fecal cultures of FF-slow degrading donors had a microbial pattern for the most abundant microbial families that approached that of FF-fast degraders. In spite of this, the absence of 2′FL degradation in these cultures points to the lack, both in the probiotic combination and the infant’s microbiota of other microorganisms, that can contribute to the efficient degradation of this HMO, as already commented on. Although lactobacilli have not been proven to ferment 2′FL efficiently in vitro [4,34], they can grow in lactose, and some species, including *L. helveticus*, can utilize the galactose moiety released from this disaccharide [35,36]. Data obtained from our fecal cultures indicate an active consumption of lactose, glucose, and galactose and a certain accumulation of fucose in 2′FL fast degrader donors during incubation, supporting an active microbial metabolism in these cultures. The *L. helveticus* R0052 strain, as well as some of the primary colonizers of the infant gut, among which lactobacilli are included, have the capacity to produce high levels of lactic acid [37], whereas some lactobacilli can also produce acetic acid at considerably lower levels [38]. Thus, the marked reduction of acetic acid concentrations, together with the increase of lactic acid levels, points to the enhancement of the metabolism of lactobacilli in cultures with added *L. helveticus*. The reduction of pyruvate levels, an intermediate metabolite of several metabolic routes in most microorganisms, supports the activation of the microbial metabolism in cultures with added *L. helveticus*. It is also worth mentioning that although the production of succinic acid by the infant’s gut microbiota has been associated in previous studies with the Enterobacteriaceae family [7,39,40], *L. helveticus* and other lactobacilli can also produce some amounts of this metabolite [38,41] which supports the involvement of lactobacilli in shifts for succinic acid occurring in fecal cultures incubated in the presence of *L. helveticus*.

Fecal cultures with the probiotic formulation added displayed a similar trend of microbiota and metabolite profiles as in cultures with added *L. helveticus* R0052, corroborating the predominant influence of this microorganism over *Bifidobacterium* strains on the fecal microbial community during incubation. In this regard, the higher proportion of *L. helveticus* than of *Bifidobacterium* strains in the probiotic formulation as well as in fecal cultures inoculated with each of the probiotic strains individually could have also favored the apparently more predominant influence of lactobacilli over the bifidobacteria strains on the microbiota. The predominant influence of *L. helveticus* R0052 over *Bifidobacterium* strains could have been probably linked to its higher level in the probiotic formulation and the higher capacity to produce organic acids (mainly lactic acid) in cultures, which may affect bifidobacteria, given that these microorganisms are generally more sensitive to acidic conditions than *L. helveticus*. These factors could have also accounted for the absence of 2′FL degradation in fecal cultures of slow degraders added with the multi-strain probiotic formulation in spite of the presence of *B. bifidum* R0071, a strain that, added alone, promoted the degradation of 2′FL.

Some of the beneficial effects of intestinal microorganisms have been associated with the production of SCFAs [28]. Among these metabolites, the most abundant by far in our study were acetic and lactic acids, these also being the most abundant microbial compounds commonly found in infant feces [8,37]. Several studies indicate that intestinal bifidobacteria can protect from enteropathogenic infection through the production of acetic acid [42]. The consumption of HMOs and the levels of intestinal bifidobacteria and acetic acid in early life were positively associated with the prevention of later respiratory tract infections in children [43]. On the other hand, acetic acid can directly promote regulatory T-cell generation in the colon [44], providing a possible role for this metabolite in the development of the mucosal immune system and the further prevention of allergies in children [45]. The intestinal microbiota has not been analyzed in the clinical trials carried out until present to prove the efficacy of the multi-strain probiotic formulation for pediatric populations. Nevertheless, Kuugbee et al. have reported a beneficial modulation of the intestinal microbiota, the improvement of intestinal mucosa barrier function, and a decrease of inflammation by the administration of the commercial formulation Biostime^®^ in a rat model of colon cancer [46].

It is tempting to speculate that some of the beneficial effects reported in such studies, such as the increase of the efficacy of standard diarrhea treatments, reduction of infections, and enhancement of the immune system [17], may be partly mediated by shifts in SCFAs, mainly acetic acid, linked to its production by the intestinal microbiota. This, in turn, suggests a possible modification in the regulation of mitochondrial-associated energy pathways of the host [47]. Lactic acid produced actively by intestinal bacteria plays an important role in the infant gut by helping to build the intestinal microbial trophic chain in the intestinal fermentation process of carbohydrates [37]. The microbial cross-feeding mechanism for lactate degradation could exert beneficial effects by increasing the production of SCFA or detrimental effects by the accumulation of gas as hydrogen and H_2_S (related with infant colic and several other conditions), depending on the microbial communities participating in the process [37,48]. At present, there is an absence of clinical data that may support the possible utility of the probiotic formulation tested in the present work to improve health conditions associated with lactic acid in the infant’s gut. However, it could be of interest to investigate whether changes promoted by this multi-strain probiotic formulation in the metabolic activity of the infant’s microbiota may exert any effect on health conditions related to the intestinal lactate.

An important limitation of our study is the low number of infant donors and high standard deviation obtained for some analyses, which precludes us from reaching sound conclusions. It should also be taken into account that some of the beneficial effects attributed to probiotics in healthy infants could not be mediated by microbial compounds and occur without apparent alterations of the intestinal microbiota community structure [49]. The results presented here support the interest to accomplish further studies intended to link the efficacy of this probiotic formulation with the microbiota profile of recipient infants.

## 5. Conclusions

A probiotic formulation and each of the constituting strains *Lactobacillus helveticus* Rosell^®^-52 (R0052), *Bifidobacterium longum* subsp. *infantis* Rosell^®^-33 (R0033), and *Bifidobacterium bifidum* Rosell^®^-71 (R0071) showed in fecal cultures of infants at two months of age, incubated in the presence of 2′FL, a differential effect on the intestinal microbiota composition and metabolic activity that was dependent on the specific probiotic strain and the intrinsic capacity of the microbiota of fecal donors to degrade 2′FL. *B. bifidum* R0071 was the sole microorganism of this probiotic formulation that was able to promote a partial increase of 2′FL consumption in cultures of 2′FL slow-degrading donors, whereas the strain *L. helveticus* R0052 and the probiotic formulation promoted changes in the microbiota profiles of formula-fed slow degraders that approached that of fast degraders. In spite of this, the inability to reach the same efficiency of 2′FL degradation as in the cultures of fast degraders point to the lack of specific microorganisms, both in the probiotic combination and the microbiota of slow-degrading donors, that could contribute efficiently to improve the consumption of 2′FL. Further studies are needed to decipher to what extent the intestinal microbiota and its intrinsic capacity to degrade 2′FL contribute to the health-promoting effects of this probiotic combination.

## Figures and Tables

**Table 1:** Family-level of the most abundant microbial taxa (relative abundance > 1% for any of the fecal culture groups) displaying significant differences at 24 h of incubation in cultures supplemented with 2′FL with and without probiotics added. Results highlighted in grey correspond to cultures with different 2′FL velocities of degradation but the same mode of feeding as the group being analyzed and were considered for statistical comparisons with the group indicated. Different letters at the right of numerical values indicate significant differences (*p* < 0.05) among conditions analyzed separately for each fecal culture group. Values for fast and slow-degrading cultures with 2′FL added without probiotics were published previously [7] and are used here for statistical comparisons only. Family names are abbreviated by removing the termination “aceae” from the taxonomic designation.

Group	Condition	Bifidobacteri-	Lactobacill-	Streptococc-	Lachnospir-	Ruminococc-	Erysipelotrich-
BF-Fast	Fast 2′FL	23.77 ± 8.48 **b**	13.08 ± 14.54 **a**	0.18 ± 0.11	0.35 ± 0.25	0.07 ± 0.09 **b**	0.01 ± 0.01 **a**
	Slow 2′FL	2.57 ± 4.23 **a**	14.99 ± 25.63 **a**	0.62 ± 0.83	2.88 ± 4.29	0.72 ± 0.72 **c**	2.35 ± 1.35 **b**
	*B.bifidum +* 2′FL	22.74 ± 10.83 **b**	12.65 ± 13.81 **a**	0.13 ± 0.04	0.44 ± 0.43	0.09 ± 0.08 **b**	0.06 ± 0.07 **a**
	*B.infantis +* 2′FL	19.56 ± 9.54 **b**	15.43 ± 17.35 **a**	0.10 ± 0.05	0.43 ± 0.34	0.12 ± 0.12 **b**	0.13 ± 0.22 **ab**
	*L.helveticus +* 2′FL	10.77 ± 2.76 **a**	38.16 ± 1.3 **b**	0.14 ± 0.05	0.14 ± 0.08	0.00 ± 0.01 **a**	0.12 ± 0.28 **a**
	Combination + 2′FL	11.81 ± 4.94 **a**	37.39 ± 2.63 **b**	0.10 ± 0.07	0.21 ± 0.16	0.07 ± 0.08 **b**	0.10 ± 0.13 **a**
BF-Slow	Fast 2′FL	23.77 ± 8.48 **b**	13.08 ± 14.54 **a**	0.18 ± 0.11	0.35 ± 0.25	0.07 ± 0.09 **a**	0.01 ± 0.01 **a**
	Slow 2′FL	2.57 ± 4.23 **a**	14.99 ± 25.63 **ab**	0.62 ± 0.83	2.88 ± 4.29	0.72 ± 0.72 **b**	2.35 ± 1.35 **b**
	*B.bifidum +* 2′FL	1.81 ± 1.30 **ab**	0.61 ± 0.50 **a**	2.45 ± 1.55	7.11 ± 10.35	2.38 ± 2.92 **b**	3.63 ± 1.35 **b**
	*B.infantis +* 2′FL	2.11 ± 0.43 **ab**	0.18 ± 0.09 **a**	0.18 ± 0.09	0.62 ± 0.34	0.18 ± 0.09 **ab**	4.20 ± 1.19 **b**
	*L.helveticus +* 2′FL	0.07 ± 0.02 **a**	48.25 ± 5.83 **b**	0.10 ± 0.09	0.16 ± 0.12	0.03 ± 0.02 **a**	0.91 ± 0.24 **ab**
	Combination + 2′FL	0.90 ± 1.21 **a**	45.43 ± 5.95 **b**	0.26 ± 0.3	0.48 ± 0.57	0.17 ± 0.18 **ab**	1.04 ± 0.16 **ab**
FF-Fast	Fast 2′FL	7.78 ± 0.56	45.72 ± 5.14 **b**	0.04 ± 0.01 **a**	0.03 ± 0.03 **a**	0.01 ± 0.01	0.18 ± 0.03 **bc**
	Slow 2′FL	8.02 ± 1.27	8.67 ± 9.09 **a**	31.26 ± 2.43 **b**	0.52 ± 0.20 **b**	0.02 ± 0.02	0.03 ± 0.02 **a**
	*B.bifidum +* 2′FL	8.50 ± 1.03	44.86 ± 0.83 **b**	0.03 ± 0.03 **a**	0.03 ± 0.02 **a**	0.02 ± 0.01	0.22 ± 0.02 **c**
	*B.infantis +* 2′FL	8.94 ± 1.32	38.67 ± 4.67 **ab**	0.10 ± 0.09 **a**	0.89 ± 1.46 **ab**	0.22 ± 0.38	0.19 ± 0.05 **bc**
	*L.helveticus +* 2′FL	6.18 ± 0.89	42.19 ± 2.15 **b**	0.11 ± 0.05 **ab**	0.06 ± 0.03 **ab**	0.02 ± 0.03	0.15 ± 00 **abc**
	Combination + 2′FL	5.95 ± 2.3	43.70 ± 4.81 **ab**	0.12 ± 0.03 **ab**	0.04 ± 0.04 **a**	0.01 ± 0.01	0.09 ± 0.03 **ab**
FF-Slow	Fast 2′FL	7.78 ± 0.56 **b**	45.72 ± 5.14 **b**	0.04 ± 0.01 **a**	0.03 ± 0.03 **a**	0.01 ± 0.01	0.18 ± 0.03
	Slow 2′FL	8.02 ± 1.27 **b**	8.67 ± 9.09 **a**	31.26 ± 2.4 **b**	0.52 ± 0.20 **b**	0.02 ± 0.02	0.03 ± 0.02
	*B.bifidum +* 2′FL	8.03 ± 1.82 **b**	6.78 ± 6.86 **a**	31.89 ± 3.06 **b**	0.68 ± 0.3 **c**	0.04 ± 0.05	0.02 ± 0.03
	*B.infantis +* 2′FL	8.17 ± 1.09 **b**	7.31 ± 7.82 **a**	32.09 ± 2.35 **b**	0.54 ± 0.23 **b**	0.02 ± 0.02	0.02 ± 0.04
	*L.helveticus +* 2′FL	2.86 ± 0.97 **a**	49.58 ± 7.69 **b**	12.76 ± 5.73 **a**	0.42 ± 0.42 **abc**	0.01 ± 0.02	0.02 ± 0.04
	Combination + 2′FL	2.13 ± 0.69 **a**	52.34 ± 4.58 **b**	9.87 ± 5.88 **a**	0.27 ± 0.17 **b**	0.01 ± 0.01	0.01 ± 0.02

## Data Availability

The raw sequences reported in this article were deposited in the NCBI Sequence Read Archive (SRA) under the accession number PRJNA731876. Other additional data presented in this study are available upon reasonable request from the corresponding authors.

## Supplementary Materials

The following supporting information can be downloaded at: https://www.mdpi.com/article/10.3390/microorganisms10020318/s1, Figure S1: Variations in the levels of fucose, lactose, galactose, and glucose after 24 h of incubation in fecal cultures with added 2′FL with and without the different probiotics and the probiotic formulation. Table S1: Description of independent pH-uncontrolled fecal batch fermentation combinations. Table S2: Cumulative gas production, pH decrease, and variations in the levels of formic, butyric, isobutyric and isovaleric acids, and ethanol after 24 h of incubation in fecal cultures with added 2′FL, with and without the different probiotics and the probiotic formulation.
