# Supplementary material for: In Vitro Probiotic Modulation of the Intestinal Microbiota and 2′Fucosyllactose Consumption in Fecal Cultures from Infants at Two Months of Age
Source: Microorganisms. 2022 Jan 29;10(2):318. doi: 10.3390/microorganisms10020318 (PMC8876326; doi:10.3390/microorganisms10020318)
Supplement: Supplementary file 1 [file microorganisms-10-00318-s001.zip › Table S1.pdf]

Table S1. Independent pH-uncontrolled fecal batch fermentation combinations**.** The dashed line indicates that no probiotic or 2’FL has been added.

**Name**	**Probiotic strains**	**2’FL commercial formulation**	**Fecal samples**
L. helveticus + 2´FL	*L. helveticus* R0052	2’FL-A<br>2’FL-B<br>2’FL-C	Breastfed fast degrader (n=2)<br>Breastfed slow degrader (n=1)<br>Formula fed fast degrader (n=1)<br>Formula fed slow degraders (n=2)
L. helveticus	*L. helveticus* R0052	------	
B. infantis + 2´FL	*B. longum* subsp. *infantis* R0033	2’FL-A<br>2’FL-B<br>2’FL-C	
B. infantis	*B. longum* subsp. *infantis* R0033	------	
Probiotic formulation + 2´FL	Multi-strain mix *L. helveticus* R0052/*B. longum* subsp. *infantis* R0033/*B. bifidum* R0071,<br>Proportions 80:10:10, respectively	2’FL-A<br>2’FL-B<br>2’FL-C	
Probiotic formulation	Multi-strain mix *L. helveticus* R0052/*B. longum* subsp. *infantis* R0033/*B. bifidum* R0071,<br>Proportions 80:10:10, respectively	------	
2´FL	Negative control	2’FL-A<br>2’FL-B<br>2’FL-C	
Control	Negative control	------	
