# Supplementary material for: In Vitro Probiotic Modulation of the Intestinal Microbiota and 2′Fucosyllactose Consumption in Fecal Cultures from Infants at Two Months of Age
Source: Microorganisms. 2022 Jan 29;10(2):318. doi: 10.3390/microorganisms10020318 (PMC8876326; doi:10.3390/microorganisms10020318)
Supplement: Supplementary file 1 [file microorganisms-10-00318-s001.zip › Table S2.pdf]

Table S2. Cumulative gas production and pH decrease at 24 h of incubation as well as variations with respect to baseline (time 0) in the levels of microbial metabolites not displaying significant difference (formic, butyric, isobutyric and isovaleric acids, and ethanol) between fecal cultures added with 2′FL and probiotics and the corresponding cultures only added with 2′FL. Fecal culture groups are established according to the feeding type of donors and the intrinsic capacity of the basal microbiota to degrade 2′FL [7]: FF-fast degrader (n=1), FF-slow degraders (n=2), BF-fast degraders (n=2), BF-slow degrader (n=1). Cultures with the three commercial 2′FL formulations were considered as triplicates for a given experimental condition with the same fecal inocula. Different letters at the right of numerical values indicate significant differences ($p < 0.05$) among different conditions analyzed separately in cultures of FF and BF infants fast and-slow degraders. Results highlighted in grey correspond to fecal cultures with different 2′FL velocity of degradation but with the same mode of feeding, and were considered for statistical comparisons with the group indicated. Values for fast and slow fermenter cultures added with 2′FL without probiotics have been published previously [7] and were used here for statistical comparisons only.

Group	Condition	Cummulative gas (mL)	ΔpH	Isobutyric (Δμg/mL)	Butyric (Δμg/mL)	Isovaleric (Δμg/mL)	Propionic (Δμg/mL)	Ethanol (Δmg/100mL)	Formic (Δmg/100mL)
FF-Fast	Fast 2'FL	11.04 ± 1.55	−2.68 ± 0.02$^{\mathbf{ab}}$	0.00 ± 0.00	0.41 ± 0.90	−10.79 ± 7.46	0.00 ± 0.00	36.88 ± 24.91$^{\mathbf{ab}}$	0.06 ± 0.54$^{\mathbf{b}}$
	Slow 2'FL	9.43 ± 1.25	−2.49 ± 0.16^{a}	0.06 ± 0.14	−2.24 ± 2.57	−10.44 ± 7.24	4.92 ± 5.82	−28.71 ± 44.54^{a}	1.76 ± 0.74^{b}
	B.bifidum 2'FL	10.84 ± 0.45	−2.66 ± 0.03$^{\mathbf{a}}$	0.00 ± 0.00	1.23 ± 0.54	−6.11 ± 7.17	−0.13 ± 0.23	54.40 ± 51.46$^{\mathbf{b}}$	−0.30 ± 0.10$^{\mathbf{ab}}$
	B.infantis 2'FL	10.15 ± 0.60	−2.69 ± 0.03$^{\mathbf{ab}}$	0.00 ± 0.00	0.92 ± 0.22	−9.92 ± 5.36	0.00 ± 0.00	7.30 ± 15.52$^{\mathbf{ab}}$	−0.40 ± 0.42$^{\mathbf{ab}}$
	L.helveticus 2'FL	8.91 ± 0.83	−2.83 ± 0.03$^{\mathbf{b}}$	0.00 ± 0.00	0.03 ± 0.05	−8.15 ± 7.21	0.00 ± 0.00	150.62 ± 174.28$^{\mathbf{b}}$	−3.21 ± 0.22$^{\mathbf{a}}$
	Probiotic formulation 2'FL	9.01 ± 1.05	−2.85 ± 0.02$^{\mathbf{b}}$	0.00 ± 0.00	0.18 ± 0.18	−10.07 ± 6.84	0.00 ± 0.00	52.11 ± 68.15$^{\mathbf{ab}}$	−3.73 ± 0.68$^{\mathbf{a}}$
FF-Slow	Fast 2'FL	11.04 ± 1.55	−2.68 ± 0.02^{b}	0.00 ± 0.00	0.41 ± 0.90	−10.79 ± 7.46	0.00 ± 0.00	36.88 ± 24.91	0.06 ± 0.54
	Slow 2'FL	9.43 ± 1.25	−2.49 ± 0.16$^{\mathbf{a}}$	0.06 ± 0.14	−2.24 ± 2.57	−10.44 ± 7.24	4.92 ± 5.82	−28.71 ± 44.54	1.76 ± 0.74
	B.bifidum 2'FL	9.50 ± 1.10	−2.50 ± 0.16$^{\mathbf{a}}$	0.00 ± 0.00	−2.41 ± 1.26	−8.11 ± 2.01	3.49 ± 2.17	−7.21 ± 69.43	1.66 ± 0.51
	B.infantis 2'FL	9.44 ± 1.22	−2.52 ± 0.14$^{\mathbf{a}}$	0.00 ± 0.00	−3.07 ± 1.65	−8.72 ± 2.06	3.49 ± 3.17	−3.72 ± 88.73	2.73 ± 0.99
	L.helveticus 2'FL	8.56 ± 1.66	−2.81 ± 0.15$^{\mathbf{b}}$	0.00 ± 0.00	−3.71 ± 4.11	−10.65 ± 3.23	2.94 ± 2.40	46.72 ± 65.18	2.62 ± 2.40
	Probiotic formulation 2'FL	8.02 ± 1.78	−2.82 ± 0.15$^{\mathbf{b}}$	0.00 ± 0.00	−3.24 ± 2.88	−12.43 ± 3.90	3.26 ± 2.14	37.27 ± 74.60	1.38 ± 1.74
BF-Fast	Fast 2'FL	10.99 ± 4.14	−2.49 ± 0.07$^{\mathbf{a}}$	0.87 ± 2.13$^{\mathbf{a}}$	0.00 ± 0.00$^{\mathbf{a}}$	−14.34 ± 4.26	−1.15 ± 1.28	−1.97 ± 34.95	6.79 ± 7.35
	Slow 2'FL	11.80 ± 0.31	−1.80 ± 0.03^{a}	45.72 ± 11.36^{b}	2.80 ± 1.13^{b}	−7.55 ± 8.77	−2.76 ± 2.81	−21.32 ± 18.51	19.14 ± 5.26
	B.bifidum 2'FL	12.42 ± 0.60	−2.49 ± 0.07$^{\mathbf{a}}$	0.30 ± 0.75$^{\mathbf{a}}$	0.00 ± 0.00$^{\mathbf{a}}$	−10.82 ± 2.88	−0.42 ± 1.58	2.79 ± 78.83	5.78 ± 5.04
	B.infantis 2'FL	12.32 ± 1.26	−2.49 ± 0.07$^{\mathbf{a}}$	0.04 ± 0.10$^{\mathbf{a}}$	0.00 ± 0.00$^{\mathbf{a}}$	−11.03 ± 2.59	−0.71 ± 0.91	−0.53 ± 53.50	6.44 ± 6.26
	L.helveticus 2'FL	9.16 ± 0.48	−2.72 ± 0.08$^{\mathbf{b}}$	0.16 ± 0.38$^{\mathbf{a}}$	0.00 ± 0.00$^{\mathbf{a}}$	−11.88 ± 3.55	−1.00 ± 1.58	17.56 ± 26.29	5.41 ± 6.92
	Probiotic formulation 2'FL	9.60 ± 0.69	−2.75 ± 0.08$^{\mathbf{b}}$	0.53 ± 1.29$^{\mathbf{a}}$	0.00 ± 0.00$^{\mathbf{a}}$	−13.55 ± 4.05	−1.07 ± 1.93	0.25 ± 28.75	4.31 ± 5.01
BF-Slow	Fast 2'FL	10.99 ± 4.14	−2.49 ± 0.07^{b}	0.87 ± 2.13^{a}	0.00 ± 0.00^{a}	−14.34 ± 4.26^{ab}	−1.15 ± 1.28	−1.97 ± 34.95	6.79 ± 7.35
	Slow 2'FL	11.80 ± 0.31	−1.80 ± 0.03$^{\mathbf{a}}$	45.72 ± 11.36$^{\mathbf{b}}$	2.80 ± 1.13$^{\mathbf{b}}$	−7.55 ± 8.77$^{\mathbf{ab}}$	−2.76 ± 2.81	−21.32 ± 18.51	19.14 ± 5.26
	B.bifidum 2'FL	10.91 ± 3.23	−1.83 ± 0.02$^{\mathbf{a}}$	43.95 ± 6.21$^{\mathbf{b}}$	3.15 ± 1.55$^{\mathbf{b}}$	−0.98 ± 2.66$^{\mathbf{b}}$	−2.71 ± 1.92	48.85 ± 51.26	19.12 ± 2.73
	B.infantis 2'FL	13.44 ± 4.13	−2.00 ± 0.01$^{\mathbf{b}}$	38.20 ± 3.72$^{\mathbf{b}}$	2.42 ± 1.36$^{\mathbf{b}}$	−5.79 ± 1.55$^{\mathbf{b}}$	−2.71 ± 0.99	81.95 ± 100.10	17.28 ± 3.35
	L.helveticus 2'FL	8.73 ± 2.69	−2.59 ± 0.05$^{\mathbf{b}}$	5.56 ± 2.91$^{\mathbf{ab}}$	1.12 ± 0.48$^{\mathbf{ab}}$	−17.90 ± 3.49$^{\mathbf{a}}$	−2.20 ± 0.44	50.25 ± 28.46	17.11 ± 5.63
	Probiotic formulation 2'FL	10.01 ± 2.56	−2.58 ± 0.03$^{\mathbf{b}}$	6.15 ± 2.01$^{\mathbf{ab}}$	0.92 ± 0.18$^{\mathbf{ab}}$	−18.19 ± 3.08$^{\mathbf{a}}$	−1.68 ± 1.85	32.05 ± 51.19	21.11 ± 1.53
