# Supplementary figures and images for: In Vitro Probiotic Modulation of the Intestinal Microbiota and 2′Fucosyllactose Consumption in Fecal Cultures from Infants at Two Months of Age
Source: Microorganisms. 2022 Jan 29;10(2):318. doi: 10.3390/microorganisms10020318 (PMC8876326; doi:10.3390/microorganisms10020318)

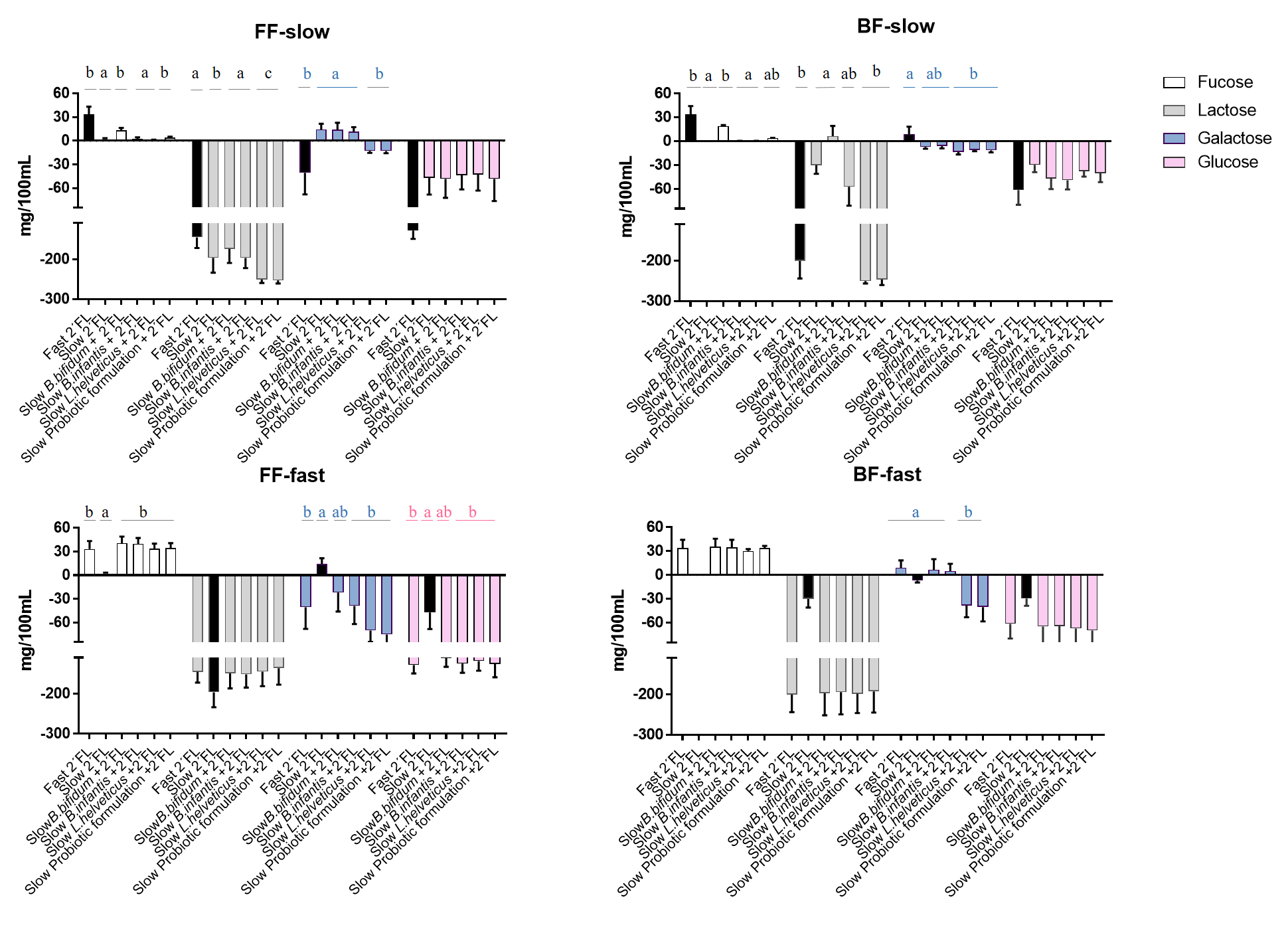

Supplement: Supplementary file 1 [file microorganisms-10-00318-s001.zip › Figure S1.BMP]
